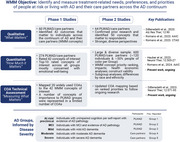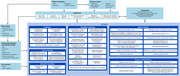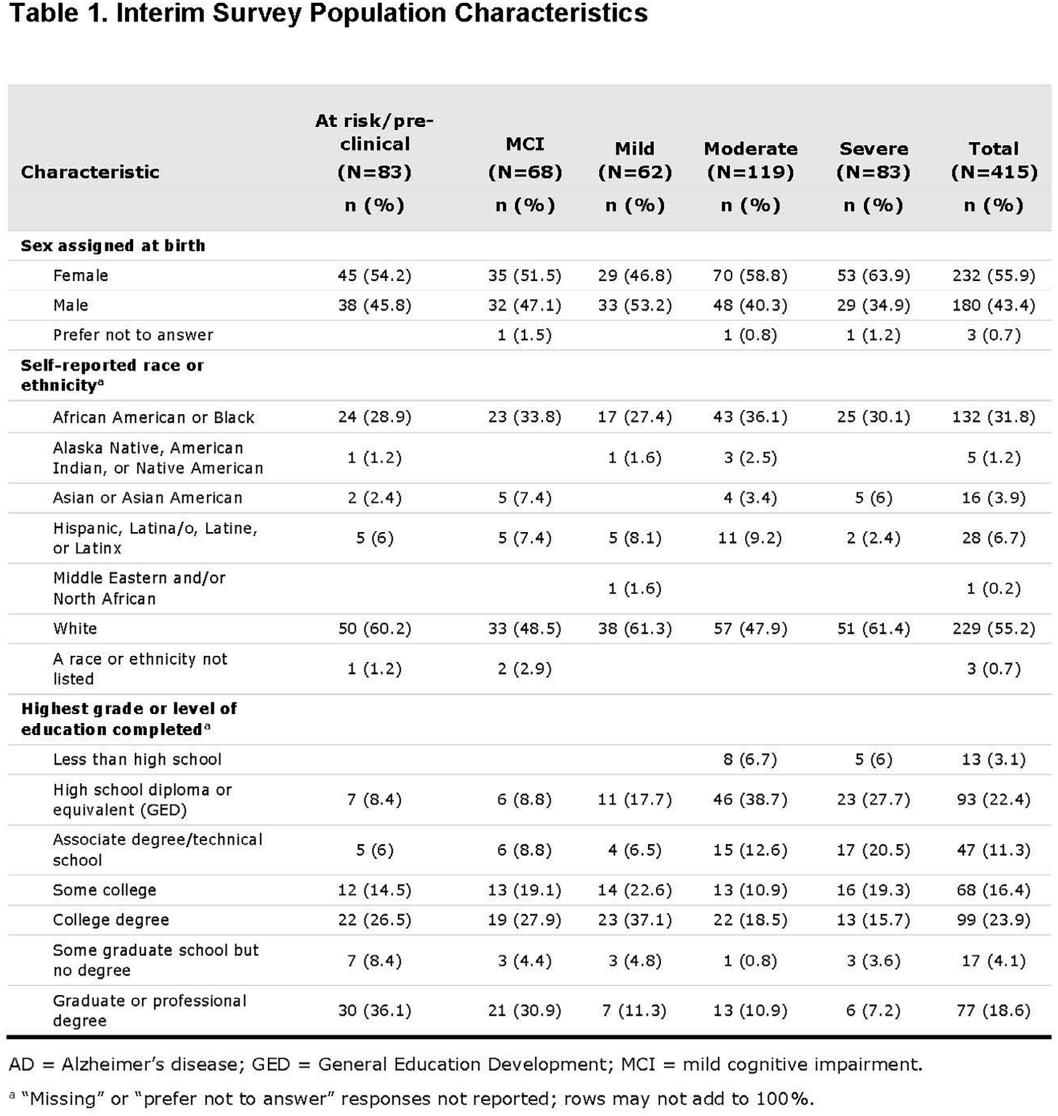# Priority Ranking: What Matters Most to People Living with Alzheimer's Disease and Care Partners

**DOI:** 10.1002/alz70858_098257

**Published:** 2025-12-24

**Authors:** Carla Romano, Emily Bratlee‐Whitaker, Ann Hartry, Jim Taylor, Leigh F Callahan, Doreen Monks, Ian Kremer, Debra Lappin, Theresa Frangiosa, Sanjyot Sangodkar, Jae Lee, Elaheh Shirneshan, Diana Slowiejko, Dana DiBenedetti, William L Herring, Kajan Gnanasakthy, Cooper Bussberg, Gabrielle Dardis, Diana Goss, Teresa Edwards, Lori McLeod, Christine Poulos, Russ Paulsen

**Affiliations:** ^1^ RTI Health Solutions, Research Triangle Park, NC, USA; ^2^ Eli Lilly and Company, Indianapolis, IN, USA; ^3^ Memory Advocate Peers (MAP), New York, NY, USA; ^4^ University of North Carolina, Chapel Hill, NC, USA; ^5^ Advocate and person living with Alzheimer's disease, Livingston, NJ, USA; ^6^ LEAD Coalition (Leaders Engaged on Alzheimer's Disease), Washington, DC, USA; ^7^ Lappin Kramer LLC, Denver, CO, USA; ^8^ Faegre Drinker Consulting, Washington, DC, USA; ^9^ AbbVie Inc., Chicago, IL, USA; ^10^ Genentech, San Francisco, CA, USA; ^11^ Karolinska Institute, Stockholm, Södermanland and Uppland, Sweden; ^12^ UsAgainstAlzheimer's, Washington, DC, USA

## Abstract

**Background:**

Considering the priorities of people living with Alzheimer's disease (PLWAD) and care partners is crucial in assessing meaningful treatment benefits for concepts encompassing symptoms, functioning, and outcomes. Previous What Matters Most (WMM) research identified and contextualized concepts confirmed as important across 5 AD Groups spanning the full spectrum of PLWAD and care partners (Figure 1). Assessing priorities among these concepts across a large, diverse population of PLWAD and care partners will further understanding of the lived experience of AD and guide development of patient‐centric study endpoints.

**Method:**

This ongoing cross‐sectional, survey‐based study will include ∼600 adults living with or at risk for AD and care partners with ∼50% people of color living in the United States. Preceding full recruitment, this interim analysis included the respondent subpopulation who completed the quantitative web‐based survey assessing prioritization of WMM concepts, clinical outcome assessments (COAs), and impacts by December 2024. Ranking was assessed using object case best‐worst scaling (BWS) questions and best‐minus‐worst scores to determine priorities and relative importance among WMM concepts within domains (Figure 2) overall and by AD Group.

**Result:**

Respondents (*n* = 415) represented diverse race and ethnicity, educational status, and AD Groups, including individuals with mild cognitive impairment or mild AD (Table 1). Overall, highest‐priority concepts by domain (alphabetically) were “Maintaining train of thought” (Communication), “Taking medications correctly” (Daily activities), “Feeling you have a sense of purpose” (Emotions), “Staying safe” (General independence), “Socializing with family” (Social life/activities), and “Understanding what others are saying in conversation” (Thought processing). Priorities differed across AD Groups. For instance, “Ability to live on your own” shifted from top‐ to middle‐priority, and the priority of “Socializing with family” increased over “Socializing with friends” for Moderate/Severe Groups compared with less‐severe Groups.

**Conclusion:**

Mixed‐methods research has confirmed the importance of WMM concepts across the AD continuum. BWS enables further contextualization through concept prioritization: interim findings suggest within‐domain priorities change over the course of disease severity. Planned full‐survey analyses include evaluation of potential demographic and clinical subgroup differences and overlaying prioritization findings with COAs and qualitative data for insights into covariance with currently experienced signs and symptoms and to support measurement of meaningful study outcomes.